# Electroacupuncture as an Adjuvant Approach to Rehabilitation during Postacute Phase after Total Knee Arthroplasty: A Systematic Review and Meta-Analysis of Randomized Controlled Trials

**DOI:** 10.1155/2021/9927699

**Published:** 2021-07-27

**Authors:** Weijian Chen, Zehua Chen, Jiao Li, Yi Wang, Guoqian Chen, Tao Jiang, Zugui Wu, Zixuan Ye, Jiayuan Zhang, Jiaxin Shan, Huai Wu, Zhen Shen, Wengang Liu, Xuemeng Xu

**Affiliations:** ^1^The Fifth Clinical Medical College of Guangzhou University of Chinese Medicine, Guangzhou 510405, China; ^2^Department of Orthopaedics, Kunming Municipal Hospital of Traditional Chinese Medicine, The Third Affiliated Hospital of Yunnan University of Chinese Medicine, Kunming, China; ^3^Department of Orthopaedic Surgery, Zhejiang Provincial Hospital of Chinese Medicine, Hangzhou, Zhejiang 310000, China; ^4^Guangdong Second Traditional Chinese Medicine Hospital, Guangzhou 510405, China

## Abstract

**Background:**

Increasing attention has been paid to electroacupuncture (EA) for promoting postoperative rehabilitation, but the effectiveness of EA for rehabilitation after total knee arthroplasty (TKA) remains obscure.

**Objective:**

To examine the effect of EA on rehabilitation after TKA.

**Methods:**

Database searches on PubMed, CINAHL, Embase, and China National Knowledge Infrastructure (CNKI) were carried out to obtain articles, from inception to 15 October 2020. All identified articles were screened, and data from each included study were extracted independently by two investigators. Meta-analysis was conducted to assess the effects of acupuncture on pain, range of knee motion, and postoperative vomiting after TKA.

**Results:**

In the current study, a total of ten randomized clinical trials were included according to the inclusion and exclusion criteria. Compared to basic treatment, EA combined with basic treatment showed a significantly greater pain reduction on 3, 7, and 14 days postoperatively after TKA. However, we found that EA had no significant improvement in enhancing the range of knee motion and decreasing the percentage of vomiting. Subgroup analysis suggested that a combination of EA and rehabilitation training was superior to rehabilitation training in pain relief, while EA combined with celecoxib capsules showed no significant difference in improving pain compared to celecoxib capsules alone.

**Conclusions:**

In the postacute phase after TKA, EA, as a supplementary treatment, could reduce postoperative pain, but no evidence supported the benefits of EA for improving ROM of knee and decreasing the ratio of vomiting. Additional high-quality and large-scale RCTs are warranted.

## 1. Introduction

Knee osteoarthritis (KOA) is characterized by the degeneration of joint cartilage, which leads to pain, swelling, dysfunction, and even joint deformity in middle-aged and elderly patients [1]. With the growing population of aging, KOA has become a global public health concern. Total knee arthroplasty (TKA) is considered as the final treatment for KOA, which is widely used to alleviate pain in the patients with advanced KOA due to the high degree of patient satisfaction. Despite its beneficial effects, most patients continue experiencing persistent moderate/severe pain and functional limitations after TKA [2]. As is reported, patients with high postoperative pain present poorer outcomes including quality of life and function during the rehabilitation process after TKA [3].

In order to allow accelerated postoperative rehabilitation, many therapeutic methods focusing on pain reduction and function improvement have been developed. As the first-line methods, pharmacological therapies such as steroids [4], opioids [5], and nonsteroidal anti-inflammatory drugs [6] are preferred in clinic practice. However, drug-related side effects like nausea, vomiting, and retention of urine [7] are frequently reported, which require prescribers to remain vigilant when prescribing the relevant drugs. Therefore, it is critical and urgent to explore a safe, effective, and feasible nonpharmacological therapy for postoperative rehabilitation to reduce the consumption of medications and related adverse effects in TKA patients [8].

Electroacupuncture (EA), as a pain management technique, has been utilized worldwide to treat acute and chronic pain. It is suggested that EA can activate various bioactive chemicals via peripheral, spinal, and supraspinal mechanisms [9]; inhibit the induction and transmission of pain signals; regulate the interactions of neuro-immune-endocrine; and consequently improve pain and inflammatory [10]. In recent years, it is proposed to be applied for rehabilitation in individuals undergoing TKA. Until now, only one meta-analysis [11] including two RCTs reported the inexplicit effect of EA on postoperative pain after TKA. However, an increasing number of studies report the impacts of EA on rehabilitation in TKA patients, and some randomized controlled trials (RCTs) aiming to evaluate the efficacy of EA for rehabilitation after TKA are being carried out [12, 13]. In this study, we undertook a systematic review and meta-analysis by gathering evidence from the available RCTs on EA to assess its effectiveness in rehabilitation for patients receiving TKA.

## 2. Methods

In this study, ethical approval was not required because all the analyses were performed according to data published in previous studies. And this meta-analysis was conducted following the Preferred Reporting Items for Systematic Review and Meta-Analyses [14].

### 2.1. Search Strategy

In order to identify relevant studies, we searched electronic databases, including Cumulative Index to Nursing and Allied Health Literature (CINAHL), PubMed, Embase, and China National Knowledge Infrastructure (CNKI) database, from inception to 15 October 2020. Keywords such as “electroacupuncture”, “total knee arthroplasty”, “total knee replacement”, “total knee^*∗*^”, “randomized controlled trial”, “controlled clinical trial”, “randomly”, “randomized”, “placebo”, “trial”, and so on were utilized to search without restrictions. The search strategy was recorded in detail in Supplementary eFigure 1. Two researchers (WJ Chen and ZH Chen) independently screened titles and abstracts. Subsequently, the remaining literatures were screened strictly by reading full texts, and all eligible studies were included according to the inclusion and exclusion criteria. Finally, the materials and data in the included studies were extracted. During the period of screening and data extraction, the discrepancy would be resolved through discussion or consultation with the primary reviewer.

### 2.2. Selection Criteria

In the current study, the PICO (patients, interventions, comparators, and outcomes) question was taken into consideration at our primary search [15]. The inclusion criteria were as follows: (1) study design: clinical randomized controlled study; (2) patients: patients receiving primary TKA; (3) intervention: EA; (4) comparators: EA versus other treatments, EA + other treatments versus other treatments, and EA versus placebo or sham EA; (5) outcomes: postoperative rehabilitation, at least one efficacy index; and (6) languages: Chinese and English. Studies would be excluded if they met any of the following criteria: (1) conference abstracts, full-text unavailable articles, or unpublished literatures and (2) repeated publications, revision TKA, unicompartmental knee arthroplasty, animal experimental studies, manual acupuncture, transcutaneous electrical stimulation, transcutaneous neuromuscular electrical stimulation, meta-analysis, or reviews.

### 2.3. Data Extraction

The two researchers who screened the literatures independently extracted the following information from the included articles: authors' names, publication year, countries involved, age and gender of patients, study design, sample size, intervention type and control characteristics, acupuncture points, needle retaining time, intervention dose, and main outcomes.

### 2.4. Quality Assessment

The quality of the included RCTs was qualitatively assessed using the risk of bias table according to 5.1.0 [16] of the Cochrane manual. The risk of bias is structured into seven aspects: sequence generation, allocation concealment, blind of participants and personnel, blind of outcome, incomplete outcome data, selective reporting, and other biases. The risk of each item is categorized into three levels: high, unclear, and low.

### 2.5. Statistical Analysis

This meta-analysis was conducted using the Review Manager 5.3 software to examine the effects of EA on postoperative rehabilitation after TKA using the reported indicators in the included literatures, and the corresponding results were depicted by the forest map intuitively. The continuous variables were pooled by standard mean differences (SMDs) or mean differences (MDs) with 95% confidence intervals (95% CI), while the odds ratios (OR) were used to estimate the enumeration data. Heterogeneity assessment was performed using Cochran's Q-test and the *I*^2^ index [17]. When *I*^2^ was statistically greater than 50%, a random-effects model would be utilized. According to the *Cochrane Handbook for Systematic Reviews of Interventions* [18], sensitivity analyses or subgroup analyses would be applied when substantially heterogeneous was detected among more than 5 studies. Begg's and Egger's tests were selected to evaluate publication bias [19]. *P* values <0.05 were viewed as statistically significant differences.

## 3. Results

### 3.1. Study Selection

**Figure 1:**
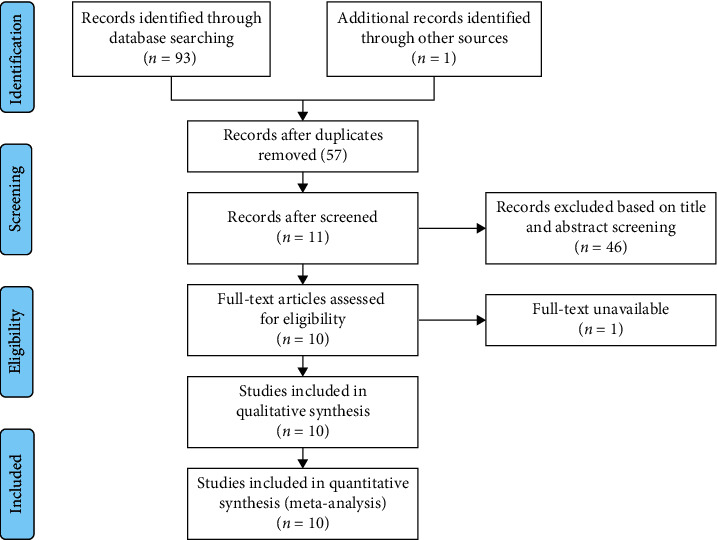
Flowchart of study selection.

A total of 94 potentially relevant records were yielded by searching Chinese and English databases. After removing 37 duplicates, eliminating 46 articles by screening titles and reading summary and full text, and excluding one study without full text (Supplementary eTable 1), 10 RCTs [20–29] were included, and 484 TKA patients with the experimental group (*n* = 241) and the control group (*n* = 243) were enrolled. The flowchart for the selection process was depicted in Figure 1, and the characteristics of each included RCT are summarized in Table 1.

### 3.2. Risk of Bias

**Figure 2:**
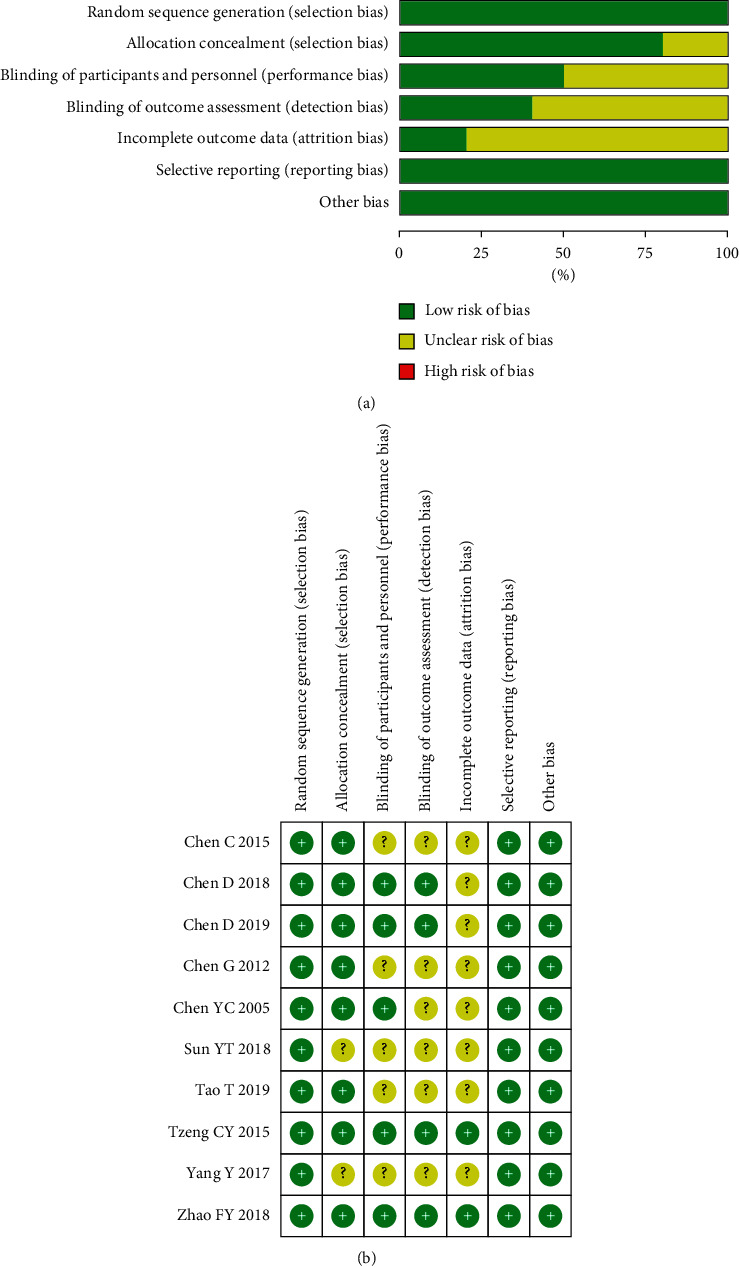
Risk of bias graph.

All the included studies were described as random generation, and 8 articles [19–24, 27, 28] documented the methods of randomization in detail. Five of the 10 included studies recorded blind methods in detail [19, 20, 22, 24, 28]. As shown in Figure 2, most of the included RCTs were defined as low risk of bias; we could conclude that the methodological quality of the included studies was fair to middling.

### 3.3. Meta-Analysis

#### 3.3.1. Postoperative Pain

**Figure 3:**
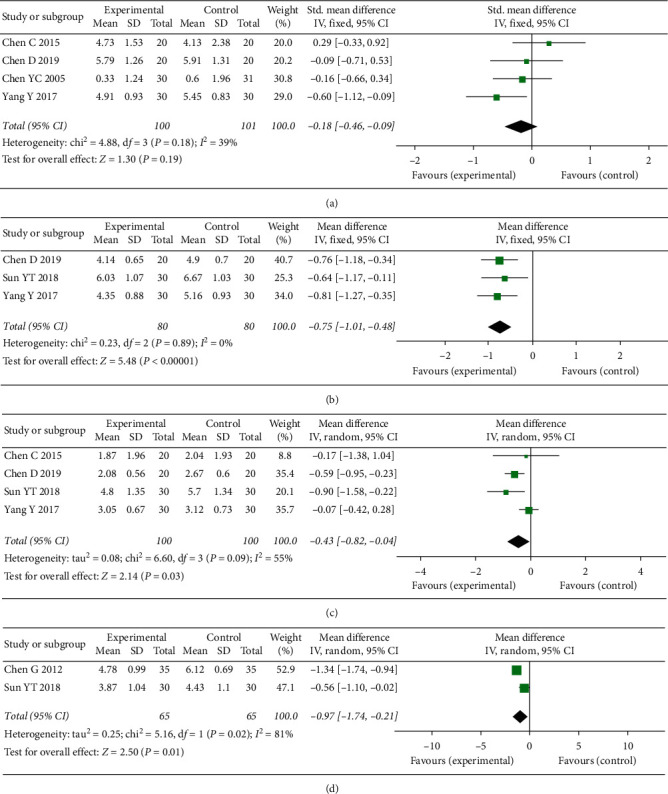
Meta-analysis and forest plot for postoperative pain at different periods. (a–d) Pain on postoperative day 1, 3, 7, and 14, respectively.

From the fixed-effects model, the meta-analysis of 4 studies [20, 27–29] suggested no statistically significant improvement in the electroacupuncture group (EG) versus the control group, with mean differences of −0.18 (95% CI: −0.46, 0.09; *P*=0.19; *I*^2^ = 39%) on pain evaluated on the first day after surgery (Figure 3(a)). However, a significantly greater pain reduction was observed in the EG when compared to the CG on postoperative day 3 (MD = −0.75; 95% CI: −1.01, −0.48; *P* < 0.00001; *I*^2^ = 0%; Figure 3(b)). Meta-analysis of 4 pain relief studies [22, 26–28] was performed using a fixed-effects model because of substantial heterogeneity, the result of which revealed a significant pain improvement in EG in comparison to the CG at 7-day follow-up (MD = −0.43; 95% CI: −0.82, −0.04; *P*=0.03; *I*^2^ = 55%; Figure 3(c)). Moreover, the EG showed a smaller VAS score than the CG on postoperative day 14 (MD = −0.97; 95% CI: −1.74, −0.21; *P*=0.01; *I*^2^ = 81%; Figure 3(d)).

#### 3.3.2. Range of Motion (ROM) and Nausea/Vomiting (an Analgesia-Related Adverse Effect)

**Figure 4:**
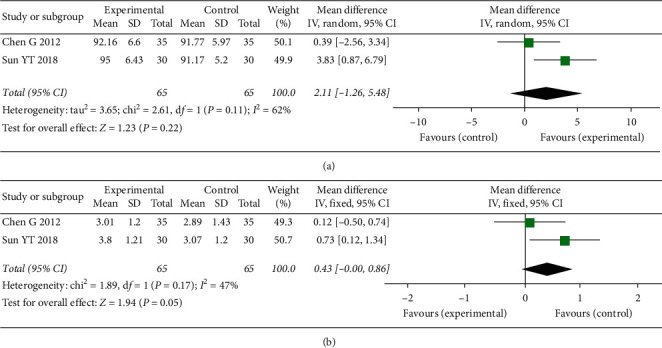
Meta-analysis and forest plot for ROM of knee. (a) Maximum flexion angle of knee; (b) active range of movement in knee extension (extension deficit).

**Figure 5:**
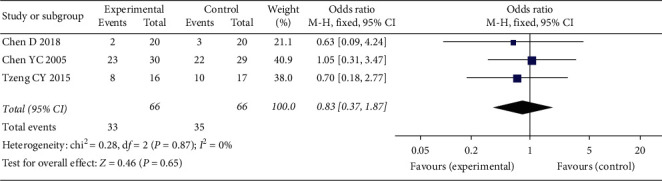
Meta-analysis and forest plot for nausea/vomiting.

ROM of knee was recorded in 3 studies [23, 24, 26], while sufficient data was not provided in 1 study [23]. We attempted to contact authors but received no response. The analysis result (Figure 4) suggested that there was no significant improvement in both flexion and extension deficit of knee between EG (MD = 2.11; 95% CI: −1.26, 5.48; *P*=0.22; *I*^2^ = 62%) and CG (MD = 0.43; 95% CI: −0.00, 0.86; *P*=0.05; *I*^2^ = 47%) at 2-week follow-up. Meanwhile, meta-analysis of 3 studies [21, 23, 29] showed there was a closely similar percentage of nausea/vomiting in the 2 groups (OR = 0.83; 95% CI: 0.37, 1.87; *P*=0.006; *I*^2^ = 0%; Figure 5).

### 3.4. Sensitivity Analysis

In this study, considering the substantial heterogeneity in postoperative pain at 7-day follow-up, we conducted sensitivity subgroup analyses to detect the source of heterogeneity. The subgroup analysis was performed according to the different comparisons, and we found that there was a significantly greater pain reduction improvement in the EG treated with a combination of EA and rehabilitation training when compared to the CG treated with rehabilitation training only (MD = −0.71; 95% CI: −1.34, −0.09; *P*=0.03; *I*^2^ = 6%). However, no significant improvement in pain between the EG treated with EA and celecoxib capsules and the CG receiving celecoxib capsules (MD = −0.33; 95% CI: −0.82, −0.44; *P*=0.21; *I*^2^ = 75%; Supplementary eFigure 2).

### 3.5. Publication Bias

In this study, we examined publication bias using Begg's and Egger's tests. As shown in Supplementary eTable 2, we found there was no evidence for significant publication bias among the included studies.

### 3.6. Adverse Events

No serious adverse event was reported in the included studies.

## 4. Discussion

EA has been proved to be beneficial for pain relief, and its unique advantages in alleviating pain are increasingly taken seriously due to few side effects. Considering its advantages, it has been commonly used for the treatment of KOA, and there are some evidence supporting the efficacy in increasing knee ROM, reducing pain, and improving function for KOA patients [30]. Postoperative pain always attracted worldwide attention, especially major orthopedic surgery, such as TKA. A substantial number of TKA patients suffer from persistent pain, and the ratio of patients with moderate-to-severe pain is up to 28%, which can strongly influence the success of postoperative rehabilitation [31]. It is crucial and urgent to find an effective method to achieve excellent pain management after surgery. It was previously reported that EA could exhibit greater analgesic effects during the treatment of different types of pain [32]. Gradually, EA began to be used to try solving this problem in the clinic. However, even though several studies reported the effect of EA on pain, ROM, and function during the recovery period, the conclusion is inconsistent. In the previous meta-analysis including 2 RCTs, there was no evidence whether EA should be recommended explicitly or not. In our study, we pooled more RCTs published before to estimate the effects of EA on rehabilitation for patients receiving TKA.

Overall, regarding postoperative pain, the results of this study revealed that EA exerted a significantly positive impact on pain control in the early postoperative phase after TKA. We found that patients treated with EA combined with the basic therapy revealed a significant pain reduction when compared to patients receiving the basic therapy alone on postoperative day 3, 7, and 14. However, EA displayed no significant improvement in pain on the first postoperative day. It might be the reason that patients receiving continuous regional anesthesia would show a lower pain within postoperative 24 hours, which resulted in an undetectable difference in pain improvement due to interferences of anesthesia resuscitation period. In our subgroup analysis of pain on postoperative day 7, our findings suggested that a combination of EA and rehabilitation training was superior to rehabilitation training alone in pain relief, while EA combined with celecoxib capsules seemed to be equal to celecoxib capsules alone in improving pain. It could be explained that celecoxib capsules, as a specific cyclooxygenase-2 inhibitor, could reach excellent pain management on day 7 after TKA so that the effect of EA on pain could not be reflected adequately. By contrast, the benefit of EA in pain improvement was more explicit when eliminating the masking effect of analgesic.

In addition, the results derived from this study revealed that EA was ineffective for improving ROM of the knee and reducing the percentage of vomiting after the operation. ROM represented joint flexion activity, which was closely associated with joint function and mobility. Previously, many studies investigated the effect of EA on ROM of various joints, while different results varied with different assessed joints. Many studies highlighted that EA could improve ROM of cervical vertebra [33], knee [34, 35], and shoulder [36], while it was proved that EA would reduce quadriceps strength, which was not good for improving knee ROM because of concomitant impaired quadriceps strength in TKA patients.

Postoperative nausea/vomiting was one of the analgesia-related adverse effects, which reduced the patient's satisfaction and consequently influenced the recovery confidence after the operation. As was known to us, EA was effective in the prevention of postoperative nausea and vomiting, the mechanism of which was considered to be related to reducing the content of 5-hydroxytryptamine and dopamine [37]. Moreover, it was reported that therapeutic outcome could be affected by acupoints, intervention point [38,39], and EA frequency [40]. In the previous studies, Neiguan (PC6) [40, 41] and 2Hz/100Hz frequency [41] were recommended to be applied in clinical practice.However, in the included studies, those intervention parameters of EA had substantial heterogeneity and were not selected as recommended. It could be the reason why the effect of EA on postoperative vomiting was not satisfactory.

There are several limitations in this study. Firstly, blind methods were not recorded in detail in most of the included studies. Secondly, the acupoints, intervention dose, and frequency selected for treatment in the 10 included studies are not consistent, which may influence the reported effects. Thirdly, we could only include a small number of studies in the analysis for several outcomes because some studies lack sufficient data. Fourthly, we can't assess the long-run effect because of the lack of RCTs with long-term follow-up. Therefore, in the future, RCTs with the long-term following and focusing on the comparison of EA combined with analgesia and analgesia used alone should be conducted to identify further the efficacy of EA on pain after TKA. Meanwhile, considering the effect of acupoint selection for outcomes, RCTs with the same acupoint selection could be more reasonable to obtain a persuasive conclusion.

## 5. Conclusion

In this paper, we systematically reviewed and quantified the effect of EA on postoperative rehabilitation for patients receiving TKA. Overall, EA, as a supplementary treatment, could reduce pain on day 3 to 14 after TKA. However, more than 7 days after TKA, this positive efficacy might not be significant when EA combined with analgesic was applied to treat postoperative pain in comparison to analgesic only. Notably, EA was found to be ineffective for improving ROM of the knee and decreasing the ratio of vomiting after surgery. But given the limitation in this study, additional high-quality and large-scale RCTs and systemic reviews are needed to confirm these findings.

## Supplementary Materials

Supplementary Materials Supplementary eFigure 1: the search strategy in this review. Supplementary eFigure 2: subgroup analysis and forest plot for postoperative pain at 7-day follow-up. Supplementary eTable 1: excluded studies found from search strategy and reason. Supplementary eTable 2: assessment of publication bias.
