# Supplementary material for: Electroacupuncture as an Adjuvant Approach to Rehabilitation during Postacute Phase after Total Knee Arthroplasty: A Systematic Review and Meta-Analysis of Randomized Controlled Trials
Source: Evid Based Complement Alternat Med. 2021 Jul 27;2021:9927699. doi: 10.1155/2021/9927699 (PMC8355970; doi:10.1155/2021/9927699)
Supplement: Supplementary Materials — Supplementary eFigure 1: the search strategy in this review. Supplementary eFigure 2: subgroup analysis and forest plot for postoperative pain at 7-day follow-up. Supplementary eTable 1: excluded studies found from search strategy and reason. Supplementary eTable 2: assessment of publication bias. [file 9927699.f1.docx]

Supplementary Material

**The search strategy used for Pubmed:**

| Query | Results |
| --- | --- |
| (((((((((((((((((((((((((((((((((Arthroplasties, Replacement, Knee) OR (Arthroplasty, Knee Replacement)) OR (Knee Replacement Arthroplasties)) OR (Knee Replacement Arthroplasty)) OR (Replacement Arthroplasties, Knee)) OR (Knee Arthroplasty, Total)) OR (Arthroplasty, Total Knee)) OR (Total Knee Arthroplasty)) OR (Replacement, Total Knee)) OR (Total Knee Replacement)) OR (Knee Replacement, Total)) OR (Knee Arthroplasty)) OR (Arthroplasty, Knee)) OR (Arthroplasties, Knee Replacement)) OR (Replacement Arthroplasty, Knee)) OR (Arthroplasty, Replacement, Partial Knee)) OR (Unicompartmental Knee Arthroplasty)) OR (Arthroplasty, Unicompartmental Knee)) OR (Knee Arthroplasty, Unicompartmental)) OR (Unicondylar Knee Arthroplasty)) OR (Arthroplasty, Unicondylar Knee)) OR (Knee Arthroplasty, Unicondylar)) OR (Partial Knee Arthroplasty)) OR (Arthroplasty, Partial Knee)) OR (Knee Arthroplasty, Partial)) OR (Unicondylar Knee Replacement)) OR (Knee Replacement, Unicondylar)) OR (Partial Knee Replacement)) OR (Knee Replacement, Partial)) OR (Unicompartmental Knee Replacement)) OR (Knee Replacement, Unicompartmental)) OR ("Arthroplasty, Replacement, Knee"[Mesh])) AND ((electroacupuncture [MeSH Terms]) OR (electroacupuncture)) AND (randomized controlled trial[Publication Type] OR randomized[Title/Abstract] OR placebo[Title/Abstract]) | **10** |

**The search strategy used for EMBASE:**

#1 'knee arthroplasty'/exp **46378**

#2 'knee arthroplasty' **46753**

#3 'arthroplasty, replacement, knee' OR 'arthroplasties, replacement, knee' OR 'arthroplasty, knee replacement' OR 'knee replacement arthroplasties' OR 'knee replacement arthroplasty' OR 'replacement arthroplasties, knee' OR 'knee arthroplasty, total' OR 'arthroplasty, total knee' OR 'total knee arthroplasty' OR 'replacement, total knee' OR 'total knee replacement' OR 'knee replacement, total' OR 'knee arthroplasty' OR 'arthroplasty, knee' OR 'arthroplasties, knee replacement' OR 'replacement arthroplasty, knee' OR 'arthroplasty, replacement, partial knee' OR 'unicompartmental knee arthroplasty' **47718**

#4 'arthroplasty, unicompartmental knee' OR 'knee arthroplasty, unicompartmental' OR 'unicondylar knee arthroplasty' OR 'arthroplasty, unicondylar knee' OR 'knee arthroplasty, unicondylar' OR 'partial knee arthroplasty' OR 'arthroplasty, partial knee' OR 'knee arthroplasty, partial' OR 'unicondylar knee replacement' OR 'knee replacement, unicondylar' OR 'partial knee replacement' OR 'knee replacement, partial' OR 'unicompartmental knee replacement' OR 'knee replacement, unicompartmental'  **968**

#5 'electroacupuncture'/exp  **6931**

#6 'electroacupuncture' **8133**

#7 #1 OR #2 OR #3 OR #4  **49374**

#8 #5 OR #6 **8133**

#9 #7 AND #8 **20**

#10 'randomized controlled trial'/exp OR 'controlled clinical trial'/exp OR 'randomized':ti,ab OR 'placebo':ti,ab OR 'drug therapy':lnk OR 'randomly':ti,ab OR 'trial':ti,ab OR 'groups':ti,ab  **7571467**

#11 #9 AND #10  **16**

**The search strategy used for CINAHL:**

#1 MeSH descriptor: [Arthroplasty, Replacement, Knee] explode all trees **2468**

#2 ((Arthroplasty, Replacement, Knee) OR (Arthroplasties, Replacement, Knee) OR (Arthroplasty, Knee Replacement) OR (Knee Replacement Arthroplasties) OR (Knee Replacement Arthroplasty) OR (Replacement Arthroplasties, Knee) OR (Knee Arthroplasty, Total) OR (Arthroplasty, Total Knee) OR (Total Knee Arthroplasty) OR (Replacement, Total Knee) OR (Total Knee Replacement) OR (Knee Replacement, Total) OR (Knee Arthroplasty) OR (Arthroplasty, Knee) OR (Arthroplasties, Knee Replacement) OR (Replacement Arthroplasty, Knee) OR (Arthroplasty, Replacement, Partial Knee) OR (Unicompartmental Knee Arthroplasty) OR (Arthroplasty, Unicompartmental Knee) OR (Knee Arthroplasty, Unicompartmental) OR (Unicondylar Knee Arthroplasty) OR (Arthroplasty, Unicondylar Knee) OR (Knee Arthroplasty, Unicondylar) OR (Partial Knee Arthroplasty) OR (Arthroplasty, Partial Knee) OR (Knee Arthroplasty, Partial) OR (Unicondylar Knee Replacement) OR (Knee Replacement, Unicondylar) OR (Partial Knee Replacement) OR (Knee Replacement, Partial) OR (Unicompartmental Knee Replacement) OR (Knee Replacement, Unicompartmental)):ti,ab,kw (Word variations have been searched) **7972**

#3 MeSH descriptor: [Electroacupuncture] explode all trees **802**

#4 (electroacupuncture):ti,ab,kw (Word variations have been searched) **2505**

#5 #1 OR #2 **7972**

#6 #3 OR #4  **2505**

#7 #5 AND #6 **16**

**The search strategy used for CNKI:**

主题=电针 or 主题= 电针 or ( 题名= 电针 or 题名=电针) (精确匹配) and 主题=膝关节置换 or ( 题名= 膝关节置换) (精确匹配) **51**

**Supplementary eFigure 1** The search strategy in this review.


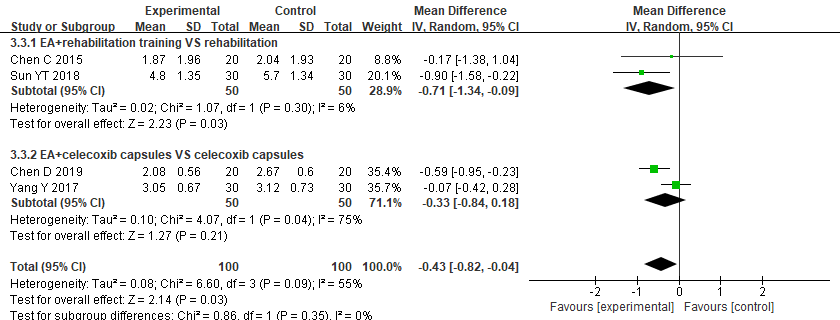


**Supplementary eFigure 2** Subgroup analysis and forest plot for postoperative pain at 7-day follow-up.

**Supplementary eTable 1** Excluded studies found from search strategy and reason

| No | First author | Excluded randomised controlled trials | Reason |
| --- | --- | --- | --- |
| 1 | Raddaoui K | **Electroacupuncture for postoperative analgesia in total knee arthroplasty**. | Full text  unavailable |

**Supplementary eTable 2** Assessment of publication bias.

| Outcomes | N | Begg' s test | Egger's test |
| --- | --- | --- | --- |
| Pain (POD 1) | 4 | 0.296 | 0.240 |
| Pain (POD 3) | 3 | 0.296 | 0.361 |
| Pain (POD 7) | 4 | 0.308 | 0.604 |
| Pain (POD 14) | 2 | 1.00 | Not available |
| ROM (flexion, POD 14) | 2 | 1.00 | Not available |
| ROM (extension, POD 14) | 2 | 1.00 | Not available |
| Ratio of nausea/vomiting | 2 | 1.00 | 0.373 |

POD: postoperative day; N: number of studies.

1. Raddaoui K, Radhouani M, Zoghlami K, et al. Electroacupuncture for postoperative analgesia in total knee arthroplasty. *Douleurs* 2019, 20(6): 291-297.
